# Supplementary material for: Campomanesia adamantium O Berg. fruit, native to Brazil, can protect against oxidative stress and promote longevity
Source: PLoS One. 2023 Nov 16;18(11):e0294316. doi: 10.1371/journal.pone.0294316 (PMC10653513; doi:10.1371/journal.pone.0294316)
Supplement: S1 Fig — (DOC) [file pone.0294316.s001.doc]

**Supporting Information**

**
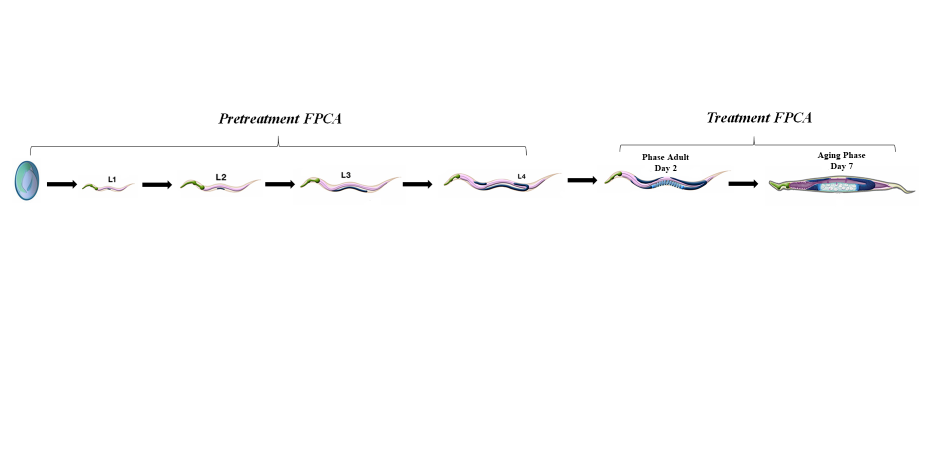
**

**S1 Fig.** **Diagram of the treatments with the *C. adamantium* fruit pulp (FPCA) during the different phases of the life cycle of *C. elegans*.**
