# Supplementary figures and images for: Campomanesia adamantium O Berg. fruit, native to Brazil, can protect against oxidative stress and promote longevity
Source: PLoS One. 2023 Nov 16;18(11):e0294316. doi: 10.1371/journal.pone.0294316 (PMC10653513; doi:10.1371/journal.pone.0294316)

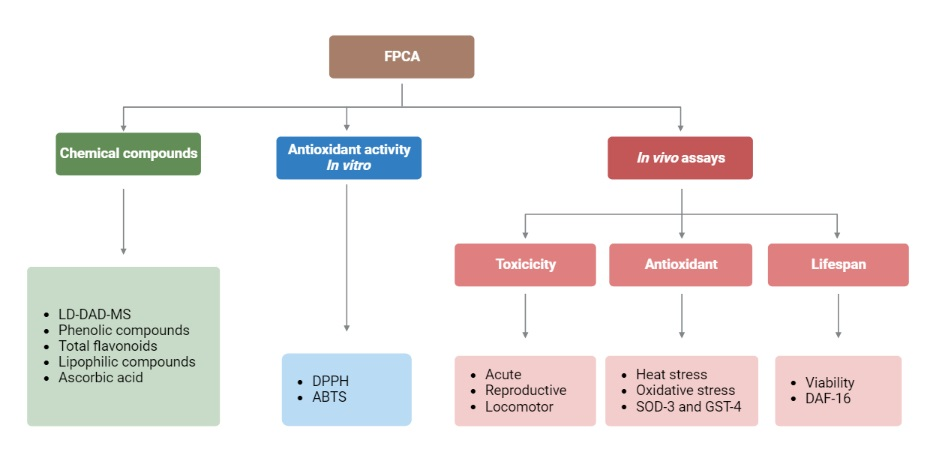

Supplement: S2 Fig — (TIF) [file pone.0294316.s002.tif]
