# Supplementary material for: Campomanesia adamantium O Berg. fruit, native to Brazil, can protect against oxidative stress and promote longevity
Source: PLoS One. 2023 Nov 16;18(11):e0294316. doi: 10.1371/journal.pone.0294316 (PMC10653513; doi:10.1371/journal.pone.0294316)
Supplement: Attachment — Submitted filename: Response to Reviewers.pdf [file pone.0294316.s003.pdf]

Response to reviewers

We are very grateful for the comments raised during the review process. All suggestions have been analyzed and the manuscript has been modified. We appreciate the suggestions in order to improve the final quality of the manuscript. Below we present our statements, and we hope they are sufficient for the requirements of PLOS ONE.

Journal Requirements:

When submitting your revision, we need you to address these additional requirements.

1. Please ensure that your manuscript meets PLOS ONE's style requirements, including those for file naming. The PLOS ONE style templates can be found at https://journals.plos.org/plosone/s/file?id=wjVg/PLOSOne_formatting_sample_main_body.pdf and https://journals.plos.org/plosone/s/file?id=ba62/PLOSOne_formatting_sample_title_authors_affiliations.pdf

Answer: The manuscript has been revised in accordance with the observations to meet the stylistic requirements as publication criteria of PLOS ONE.

2. In your Methods section, please provide additional information regarding the permits you obtained for the work. Please ensure you have included the full name of the authority that approved the field site access and, if no permits were required, a brief statement explaining why.

Answer: The *in vivo* studies described in this manuscript were conducted using the *Caenorhabditis elegans* experimental model. Ethical authorization or approval from ethics commissions or committees is not required for experimentation with this nematode.

3. Please note that PLOS ONE has specific guidelines on code sharing for submissions in which author-generated code underpins the findings in the manuscript. In these cases, all author-generated code must be made available without restrictions upon publication of the work. Please review

our guidelines at https://journals.plos.org/plosone/s/materials-and-software-sharing#loc-sharing-code and ensure that your code is shared in a way that follows best practice and facilitates reproducibility and reuse.
Answer: We have reviewed the provided link and identified an error in completing the form. However, all the research data referenced in the manuscript are readily available.

4. Thank you for stating the following in the Acknowledgments Section of your manuscript:
"This work was supported by grants from Fundação de Apoio ao Desenvolvimento da Educação, Ciência e Tecnologia do Estado de Mato Grosso do Sul (FUNDECT); Coordenação de Aperfeiçoamento de Pessoal de Nível Superior (CAPES); Conselho Nacional de Desenvolvimento Científico e Tecnológico (CNPq), Fundação de Apoio à Pesquisa no estado do Rio Grande do Sul (FAPERGS) e Financiador de Estudos e Projetos (FINEP)."

We note that you have provided funding information that is not currently declared in your Funding Statement. However, funding information should not appear in the Acknowledgments section or other areas of your manuscript. We will only publish funding information present in the Funding Statement section of the online submission form.
Please remove any funding-related text from the manuscript and let us know how you would like to update your Funding Statement. Currently, your Funding Statement reads as follows:
"The author(s) received no specific funding for this work."

Please include your amended statements within your cover letter; we will change the online submission form on your behalf.
Answer: The Acknowledgments section was changed in line 617-622 of the revised manuscript, as bellow.
"The authors would like to thank the government agencies that support science in Brazil: Fundação de Apoio ao Desenvolvimento da Educação, Ciência e Tecnologia do Estado de Mato Grosso do Sul (FUNDECT); Coordenação de

Aperfeiçoamento de Pessoal de Nível Superior (CAPES); Conselho Nacional de Desenvolvimento Científico e Tecnológico (CNPq), Fundação de Apoio à Pesquisa no estado do Rio Grande do Sul (FAPERGS) e Financiador de Estudos e Projetos (FINEP)."

5. We note that you have stated that you will provide repository information for your data at acceptance. Should your manuscript be accepted for publication, we will hold it until you provide the relevant accession numbers or DOIs necessary to access your data. If you wish to make changes to your Data Availability statement, please describe these changes in your cover letter and we will update your Data Availability statement to reflect the information you provide.
Answer: An error occurred while filling out the submission form, and no information is available to be provided to any repository. However, all the research data pertaining to the study can be found in the manuscript.

6. Please include captions for your Supporting Information files at the end of your manuscript, and update any in-text citations to match accordingly. Please see our Supporting Information guidelines for more information: http://journals.plos.org/plosone/s/supporting-information.
Answer:
The Supplementary Material caption was inserted on lines 852-854 of the revised manuscript, as below.
"Supplementary material
S1 Fig. Diagram of the treatments with the *C. adamantium* fruit pulp (FPCA) during the different phases of the life cycle of *C. elegans*."

<u>**Additional Editor Comments:**</u>
Before proceeding further kindly address following issues:
Material and methods:
Material, chemical and other components used are not mentioned in the material methodology. I must be mentioned separately in a section 2.1. Materials (make should be mentioned).

Answer: Materials, chemical components, and other components used in the manuscript's methodology were mentioned separately in the Materials section, lines 80-86 of the revised manuscript, as follows.

"The chemicals were purchased from Sigma-Aldrich: formic acid, 2,2-diphenyl-1-picrylhydrazyl, 2,2'-azinobis-(3-ethylbenzothiazoline-6-sulfonic acid, Juglone (5-hydroxy-1,4-naphthoquinone) 2,6-dichlorophenolindophenol-sodium (DCIP), potassium persulfate, butylated hydroxytoluene (BHT), quercetin, oxalic acid and sodium hypochlorite; Dinâmica: methanol, acetone, hexane, Folin-Ciocalteu, sodium carbonate, aluminum chloride hexahydrate, ascorbic acid and sodium hydroxide; Diversey: Sumaveg®."

Authors must add a flow diagram showing methodology followed in the experimentation and also show the analysis performed at each stage. This flow diagram will definitely improve the readability of the manuscript.

Answer:

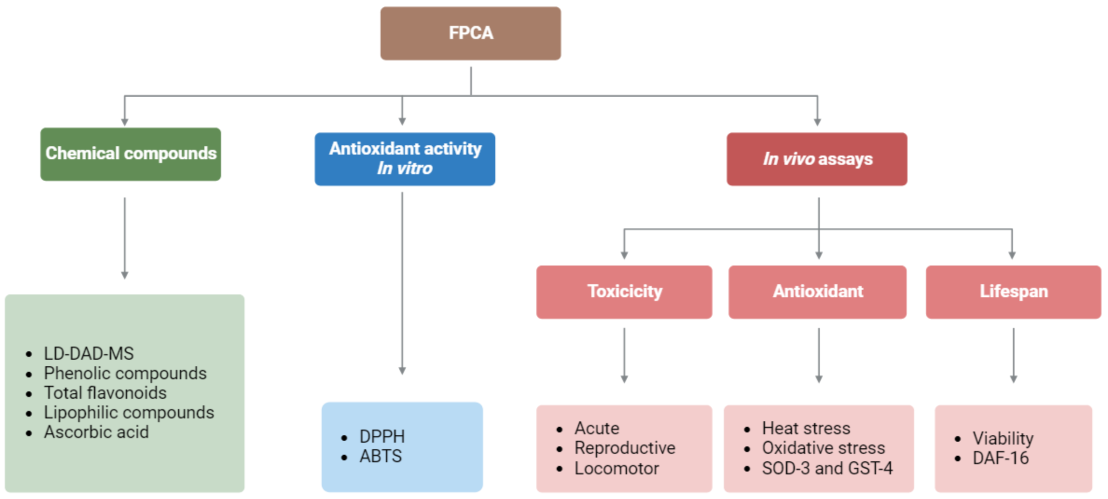

I am not able to find "conclusion" in manuscript. Please address the issues.

Answer: The conclusion is highlighted on lines 605-611 of the revised manuscript.
