# Supplementary material for: Campomanesia adamantium O Berg. fruit, native to Brazil, can protect against oxidative stress and promote longevity
Source: PLoS One. 2023 Nov 16;18(11):e0294316. doi: 10.1371/journal.pone.0294316 (PMC10653513; doi:10.1371/journal.pone.0294316)
Supplement: Attachment — Submitted filename: Response to Reviewers.docx [file pone.0294316.s005.docx]

**Response to academic editor**

We are very grateful for the comments raised during the review process. All suggestions made throughout the review process have been analyzed, and the manuscript has been modified. We appreciate the suggestions to improve the manuscript's final quality. Below we present our statements, and we hope they are sufficient for the requirements of PLOS ONE.

**ACADEMIC EDITOR:**

**I have noted the authors' considerable efforts in revising the manuscript.**

**1. However, the main body of the manuscript is lacking the inclusion of a flow chart. It is essential to incorporate the flow chart into the main body, preferably** **within the Materials and Methodology section.**

**Answer:** The flow chart was previously submitted as a response to the reviewers, but in the current version, it has been included in the main body within the Materials and Methodology section, line 98, of the revised manuscript.

**2. Additionally, the conclusion requires further elaboration in accordance with the study's findings, and it should outline potential future directions for the research.**

**Answer:** Potential future research directions have been included in the conclusion of the revised manuscript, lines 622-624.
